# Acute Inactivation of Primary Auditory Cortex Causes a Sound Localisation Deficit in Ferrets

**DOI:** 10.1371/journal.pone.0170264

**Published:** 2017-01-18

**Authors:** Katherine C. Wood, Stephen M. Town, Huriye Atilgan, Gareth P. Jones, Jennifer K. Bizley

**Affiliations:** The Ear Institute, University College London, London, United Kingdom; Universidad de Salamanca, SPAIN

## Abstract

The objective of this study was to demonstrate the efficacy of acute inactivation of brain areas by cooling in the behaving ferret and to demonstrate that cooling auditory cortex produced a localisation deficit that was specific to auditory stimuli. The effect of cooling on neural activity was measured in anesthetized ferret cortex. The behavioural effect of cooling was determined in a benchmark sound localisation task in which inactivation of primary auditory cortex (A1) is known to impair performance. Cooling strongly suppressed the spontaneous and stimulus-evoked firing rates of cortical neurons when the cooling loop was held at temperatures below 10°C, and this suppression was reversed when the cortical temperature recovered. Cooling of ferret auditory cortex during behavioural testing impaired sound localisation performance, with unilateral cooling producing selective deficits in the hemifield contralateral to cooling, and bilateral cooling producing deficits on both sides of space. The deficit in sound localisation induced by inactivation of A1 was not caused by motivational or locomotor changes since inactivation of A1 did not affect localisation of visual stimuli in the same context.

## Introduction

Manipulation of neural activity can demonstrate causal relationships between activity in a particular brain region and behavioural performance. While there are a number of methods for inactivating brain regions including physical lesions [1], pharmacological inactivation [2], optogenetics [3] and chemogenetics [4], inactivation by cooling [5] is particularly advantageous for manipulating large areas of tissue that may be intractable when using genetic tools or optical stimulation/suppression [6]. Furthermore unlike physical lesions and slow-release implantable drugs, the effects of cooling are acute and reversible [5], limiting the potential for other brain regions to compensate for the loss of neural function and allowing within-subject comparison of interleaved control and inactivation sessions [7]. Inactivation by cooling can disrupt behaviours in a variety of species [7–9] and shed light upon neural computations in sensory and decision-making systems [10–14].

The ability to determine the location of a sound source is important for humans and other animals for both survival and communication [15,16]. Sound localisation depends upon auditory cortex as lesions or inactivation of this region impair performance in approach-to-target localisation tasks in a variety of species [2,7,17–25]. Unilateral inactivation of primary auditory cortex in the ferret [2,20] and other animals (e.g. cat [23,26]) results in contralateral localisation deficits while preserving the localisation of sounds ipsilateral to the lesion or at the midline. In contrast, bilateral inactivation of auditory cortex produces deficits across space [2,20,24,25]. Deficits observed in the ferret with lesions or during pharmacological inactivation of A1 are more modest than those observed using reversible silencing by cooling in the cat [2,7,23,24] and it is unclear whether these differences arise from species, inactivation method or localisation task differences [7,23]. It is also unknown whether deficits in sound localisation following inactivation of ferret auditory cortex result from specific impairments in auditory processing or non-specific effects such as impaired motivation; dissociating such effects requires a control condition where animals localise stimuli in another sensory modality [2,24,25].

To resolve these issues, we tested the effects of reversible inactivation of ferret A1 by cooling in a sound localisation and a visual localisation task designed to control for non-specific effects of cooling. We predicted that bilateral inactivation of A1 would result in a sound localisation deficit across both sides of space whereas acute unilateral inactivation of A1 would result in a behavioural deficit only in the contralateral hemifield. In visual localisation, we predicted that performance would not be affected by auditory cortex inactivation. Since inactivation by cooling has not been performed in ferrets before, we first confirmed the effect of cooling on neural activity electrophysiologically. We observed significantly reduced neural activity when the cooling loop was held at a temperature of ≤10°C in all layers of cortex, which recovered upon reversal of cooling. We confirmed through measurements of the cortical temperature that cooling did not spread laterally outside of primary auditory cortex (temperatures necessary for cessation of firing were achieved only within 500 μm of the edge of the loop) and that beneath auditory cortex (which spans 1.5–2 mm) temperatures did not drop to a level that would impair neuronal firing. Consistent with our predictions, we observed performance deficits specific to the localisation of auditory and not visual stimuli. These data demonstrate that inactivation by cooling is a viable method for studying the function of brain areas during behaviour in the ferret.

## Methods

### Animals

Subjects were six adult pigmented ferrets (*Mustela putorius*, female; 1–4 years): Three were trained in an auditory and visual approach-to-target localisation task and subsequently implanted with cooling loops. Three were untrained animals in which the effects of cooling on cortical temperature (with cooling loop over A1 –one ferret) and neural activity (with cooling loop covering the suprasylvian gyrus–two ferrets) were measured under anaesthesia.

All ferrets were housed in groups of two to eight, with free access to high-protein food pellets and water bottles. For animals trained on the localisation task, water bottles were removed from the home cages in the afternoon on the day prior to training and replaced on the last day of a training run. On training days, ferrets received drinking water as positive reinforcement while performing a sound or visual localisation approach-to-target task. Water consumption during training was measured, and was supplemented as wet food in home cages at the end of the day to ensure that each ferret received at least 60 ml of water per kilogram of body weight daily.

Regular otoscopic examinations were carried out to ensure that both ears of the animals were clean and healthy. All experimental procedures were approved by the local ethical review committee and were carried out under licence from the UK Home Office, in accordance with the Animals (Scientific Procedures) Act 1986.

### Cooling loop and cooling apparatus

**Fig 1:**
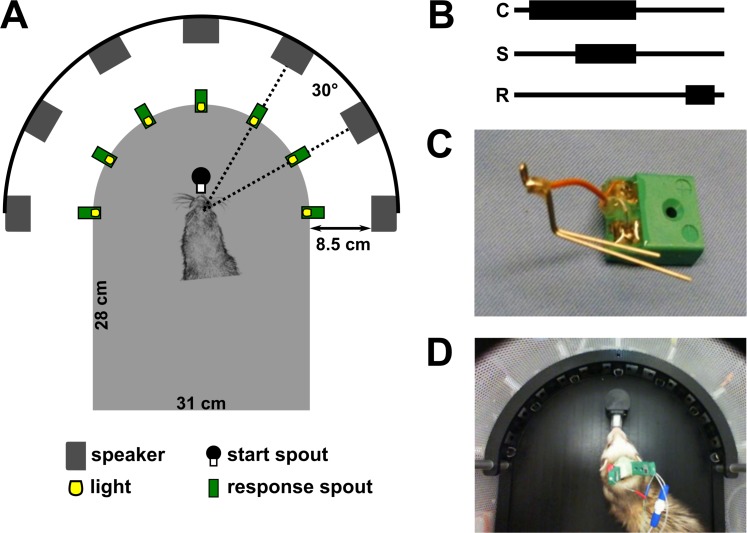
Localisation testing chamber and task outline. [A] Dimensions of the testing chamber, locations of speakers, and LEDs for the localization task [B] Task outline: the ferret must make and maintain contact with the central start spout (C) for a variable hold time before a stimulus (S, sound or light) was presented. The ferret was required to maintain contact at the start spout until the stimulus ended after which it responded (R) at one of the response spouts. [C] A cooling loop prepared for surgery with micro-thermocouple soldered and secured with epoxy acrylic to the base of the cooling loop and to a female micro-thermocouple connector. [D] Ferret with bilateral cooling loop implants, with both thermocouples and the right cooling loop connected in the localisation chamber.

The cooling loop implant was a modified, miniaturised version of the cooling loop developed by Lomber & Payne [5]. The cooling loop was constructed from 23 gauge stainless steel tubing which was bent to form a loop shape approximately the size of A1 (Fig 1C). A micro-thermocouple, made from twisting together 30 AWG gauge (0.254 mm) PFA insulated copper and constantan wire (Omega Engineering Limited, Manchester, UK), was soldered to the base of the loop and secured with an epoxy adhesive. The thermocouple wire was soldered to a miniature female thermocouple connector (RS components Ltd, UK) and again secured with an epoxy adhesive.

In each experiment the cooling loop was supplied with ethanol from a reservoir via an FMI QV drive pump (Fluid Metering, Inc., NY, USA) controlled by a variable speed controller (V300, Fluid Metering, Inc., NY, USA). Ethanol was carried in FEP and PTFE tubing (Adtech Polymer Engineering Ltd, UK) from the reservoir to pump (FEP: 1.1 mm x 2 m, inner diameter x length) and then from pump to cooling loop (FEP: 0.8 mm ID x 2 m, PTFE: 0.5 mm x 2 m). Where necessary, tubing was bridged using two-way connectors (Diba Fluid Intelligence, Cambridge, UK).

To cool the ethanol prior to arrival at the cooling loop, 1 m of PTFE tubing was coiled within a Dewar flask (Nalgene 4150–1000, NY, USA) containing a mix of ethanol (100% concentration) and dry ice (BOC, UK). To maintain ethanol temperature within the tubing system, the length of tubing between the Dewar flask and cooling loop was minimized and insulated with silicon tubing. Ethanol from the loop was recycled into the reservoir. In anesthetized experiments, cooling loops were permanently attached to the apparatus and positioned on the cortex with a Microdrive, whereas in behavioural experiments, loops were chronically implanted and connected to the apparatus prior to testing.

### Electrophysiological recordings in cortex under anaesthesia

Anaesthesia was induced by a single dose of a mixture of medetomidine (Domitor; 0.022 mg/kg/h; Pfizer) and ketamine (Ketaset; 5 mg/kg/h; Fort Dodge Animal Health). The left radial vein was cannulated and anaesthesia was maintained throughout the experiment by a continuous infusion of medetomidine (0.022 mg/kg/hr) and ketamine (5 mg/kg/hr), with atropine sulphate to reduce bronchial secretions (0.06 mg/kg/hr, C-Vet veterinary products) and dexamethasone to reduce cerebral oedema (0.5 mg/kg/hr, Dexadreson, Intervet UK) in Hartmann’s solution, supplemented with 5% glucose. The ferret was intubated, placed on a ventilator (Harvard Model 683 small animal ventilator; Harvard Apparatus) and supplemented with oxygen. Body temperature (38°C), end-tidal CO_2_, and the electrocardiogram were monitored throughout the experiment. Experiments typically lasted between 36 and 60 hours.

To access the cortex for recordings, the animal was placed in a stereotaxic frame and the temporal muscles on both sides were retracted to expose the dorsal and lateral parts of the skull. A metal bar was cemented and screwed into the right side of the skull, holding the head without further need of a stereotaxic frame. On the left side, the temporal muscle was largely removed, and a craniotomy performed to expose the suprasylvian and ectosylvian gyri. The dura was removed and the cortex covered with 1–3% agar. The animal was then transferred to a small table in a soundproof chamber (Industrial Acoustics, Winchester, UK).

Recordings were made using silicon probe electrodes (Neuronexus Technologies, Ann Arbor, MI) with either a single shank (16 channel; 100 μm site spacing). Electrodes were positioned so that they entered the cortex approximately orthogonal to the surface of the suprasylvian gyrus. Neural recordings were obtained using TDT System III hardware (RZ2 data acquisition system) with custom written software in Open Project (Tucker-Davis Technologies, Alachua, FL) and MATLAB (Mathworks, Natick, USA).

### Measuring the effects of cooling on neural responsiveness under anaesthesia

Neurophysiological validation was performed in visual cortex where spiking activity is generally higher and more robust than in auditory cortex [2]. Prior to electrode placement, we positioned the cooling loop over the surface of the suprasylvian gyrus (area posterior suprasylvian (PS) and 21, [27]). For electrode positioning and initial tests of neural responsiveness, the loop was in place, but the pump was inactive, and thus cortex remained at close to body temperature. Linear shank electrodes were then inserted such that the electrodes spanned from surface to 1.5–2 mm below the cortical surface in the centre of the cooling loop. Following a time allowed for neural activity to stabilise (typically between 5 and 30 minutes), visual responses of units in area 21 / PS were obtained in response to 100 ms flashes of white light presented using an LED housed in a diffuser placed 20° to the right of the midline of the head, 15 cm from the eye (luminance 65 cd/m^2^). Stimuli were controlled via TDT hardware (RZ6) and presented with an inter-stimulus interval of 900 ms.

Once visually responsive units were identified, the cortex was cooled by pumping chilled ethanol through the loop and adjusting the flow rate to achieve a temperature of ≤10°C (mean ± SD = 7.14 ± 1.02). During cooling, tests of visual responsiveness were repeated at regular intervals (every 5–10 minutes) for 30–80 minutes depending on the rate at which cortical temperature / visual responsiveness declined. After this period, cooling was stopped and the cortical temperature was allowed to recover for at least 25 minutes (mean ± SD: 54.5 ± 16 mins) to body temperature. During this recovery period, further tests of visual responsiveness were made.

### Mapping spread of cooling in auditory cortex

**Fig 2:**
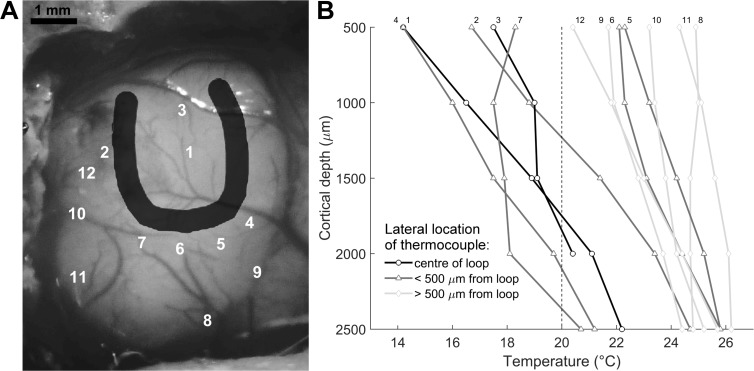
Effect of cooling on cortex temperature. [A] Locations of temperature measurements in auditory cortex with a cooling loop (dark shading) held at 10°C. At each site measurements were made at 5 depths, from 500 μm to 2500 μm below the cortical surface, in 500 μm steps. [B] Cortical temperatures at each of the positions in [A] at each depth, symbols indicate the lateral distance of the temperature recording on the cortical surface relative to the cooling loop. The dashed line shows the temperature below which neural firing ceases [5]. Ferret auditory cortex is usually ~1500 μm thick and extends to a maximum of 2000 μm at the thickest part of the gyrus [28].

In a separate series of cooling experiments performed after electrophysiological recordings were complete, we also positioned a cooling loop over primary auditory cortex and reduced the temperature of the cooling loop to 10°C. Cortical temperatures were mapped across auditory cortex by positioning a hypodermic needle temperature probe (Omega, Stamford, USA) at varying distances from the cooling loop. At each site, a micromanipulator was used to position the temperature probe in order to sample the temperature in cortex, in 500 μm steps to a depth of 2.5 mm from the cortical surface (Fig 2A).

### Electrophysiological data analysis

**Fig 3:**
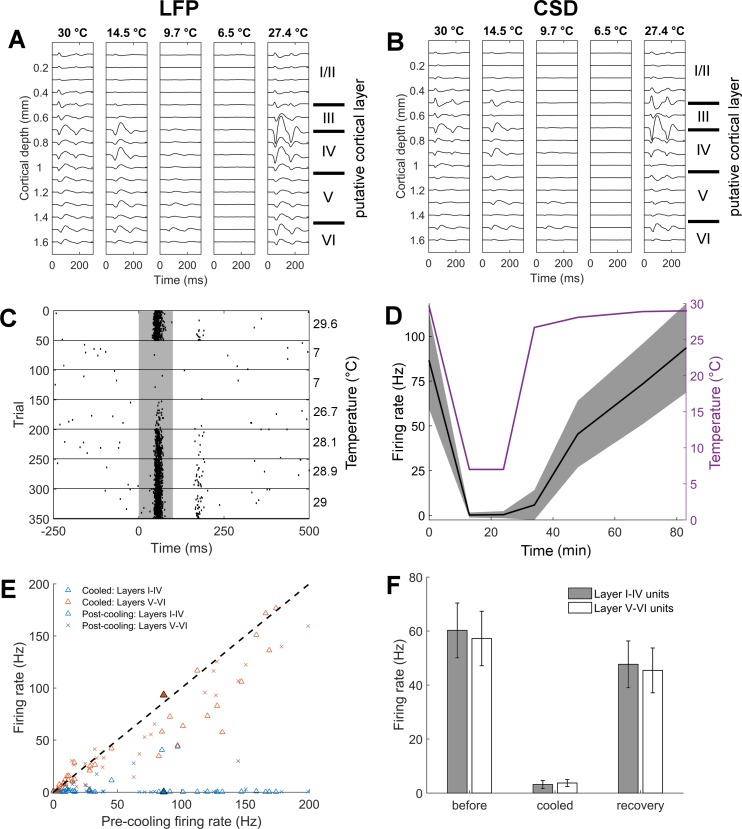
Effect of reduced cortical temperature on neuronal activity. [A] Typical light-evoked local field potentials from each electrode across the depth of the cortex for each temperature during the cooling process for a recording site in visual cortex. [B] CSD analysis of [A] indicating the locations of current sources and sinks. [C] Raster plot showing spiking activity of an example unit recorded from PS in the centre of a cooling loop before, during and after cooling in response to a 100 ms flash of white light (grey shading shows stimulus presentation). The temperature of the cooling loop during the recording is indicated. [D] Time course of mean ± SD firing rate (during 100 ms of stimulus presentation) of example multi-unit from [C]. [E] Population activity during and after cooling: Mean firing rate of superficial (triangles) and deep (crosses) units during presentation of the stimulus during (blue) and after cooling (orange) against the pre-cooling firing rate of 72 multi-units recorded from 2 ferrets (1 site and 8 sites in each ferret respectively). The example unit from [C] and [D] is shown by the filled symbols. [F] Mean activity of units from superficial (I-IV) and deep (V-VI) layers of cortex before, during and after cooling. The cooled firing rate was significantly lower than pre- and post-cooling firing rates (two-way repeated measures ANOVA, p<0.05).

For each recording site, spikes were detected online as negative deflections exceeding -3.5 times the root-mean squared signal voltage. No attempt was made to isolate single neurons as the aim here was to understand the macroscopic effects of cooling on the function of cortical populations rather than the precise properties of well-isolated single neurons. Stimulus responsive sites were defined as those at which we observed a significant evoked average local field potential (LFP) (200 ms pre-stimulus compared with 200 ms post-stimulus onset, paired t-test, p<0.05). Multi-unit spike times from responsive electrode sites were aligned relative to the time of stimulus presentation and responsive units identified as those whose spike count differed significantly during stimulus presentation compared with the 100 ms pre-stimulus baseline (paired t-test, p<0.05). The firing rate of responsive units was then plotted as a function of cortical temperature and compared between warm and cooled conditions, defined as when the temperature of the loop was 10°C or below (mean ± SD = 7.14 ± 1.02, minimum = 5.70°C). The brain was allowed to recover until it returned to within 3°C of the pre-cooling temperature (minimum 25 minutes, mean ± SD 54.5 min ± 16 min). Averaged LFP responses to the visual stimuli were monitored during each recording and analysed for changes during cooling (Fig 3A). Current source-density (CSD) analysis was applied to field potential data recorded across cortical layers using the inverse CSD method [29]. CSD analysis identifies current sinks and sources in the extracellular space and was used to estimate the layer of the recorded units and to determine changes in activity in different layers of cortex (Fig 3B).

### Behavioural task design

Ferrets were trained in a custom-built D-shaped box surrounded by an array of 7 speakers (Visaton FRS SC 5.9) at 30° intervals, 5 cm above the floor and 24 cm from the centre of the box (Fig 1A). A response spout located in front of each speaker (15.5 cm from the centre of the box and 8.5 cm in front of the speaker) contained an infra-red detector to measure the animal’s presence and provided water rewards. Above each spout was a white LED that provided light stimuli for the visual localisation task. For two ferrets (F1202 and F1204) LEDs were positioned at the same distance as the speakers (i.e. 24 cm from the centre of the chamber) whereas for one ferret (F1311) LEDs were positioned at the same distance as the response spouts (15.5 cm from the centre of the chamber, 10 cm height). A further spout was also placed at the centre of the arena to initiate stimulus presentation. This spout was offset from the centre by 3 cm to ensure the animal’s head was aligned at the centre of the speaker and LED arrays with the interaural axis in line with the -90° and 90° speakers. Outside the training box, an additional LED (15 cm from the floor) was used to indicate trial availability. The behavioural task, data acquisition, and stimulus generation were all automated using custom software running on personal computers, which communicated with TDT RX8 real-time signal processors.

In order to minimise rotations of the animal and thus twisting of the cooling tubing the testing arena was smaller than previous apparatus used for assessing sound localisation in this species (e.g. [30]) and speaker locations were restricted to frontal space. This allowed the behavioural testing to be fully automated and independent of any experimenter input. The testing arena was housed in a custom built sound attenuating chamber (Zephyr Products Ltd, Suffolk, UK) measuring 90 cm by 89.5 cm by 75 cm (height x width x depth) with the inner walls coated with sound attenuating foam (45 mm). Speakers were calibrated to produce a flat response from 200 Hz to 25 kHz using Golay codes, presented in an anechoic environment, to construct inverse filters [31]. All the speakers were matched for level using a microphone positioned upright at the level of the ferret head in the centre of the semi-circle. Calibrations were performed with a condenser microphone (Model 4191, Brüel and Kjær), a TDT System 3 RX8 signal processor and/or a Brüel and Kjær 3110–003 measuring amplifier.

### Stimuli

Auditory stimuli were white noise bursts of differing durations (500 ms, 250 ms or 100 ms) cosine ramped with 5-ms duration at the onset and offset and low pass filtered below 22 kHz (finite impulse response filter <22 kHz, 70 dB attenuation at 22.2 kHz). Noise bursts were generated afresh on each trial in MATLAB at a sampling frequency of 48848.125 Hz and presented from one speaker. Across trials, stimuli were presented at pseudorandom locations at one of three pseudorandomly selected intensities (57, 61.5 and 66 dB SPL). For two subjects, visual stimuli were presented using continuous pulses (2.5 V, 65 cd m^-2^) with pulse duration adjusted for each ferret to match sound localisation performance for sounds of 250 ms duration. For one ferret (F1202) visual stimuli were 500 ms long, and for the second ferret (F1311) visual stimuli were 750 ms. A further ferret was trained with visual stimuli but did not achieve consistent performance in the limited time available for testing.

### Training

Training runs were 5 days long and consisted of two training sessions per day separated by approximately 5 hours. Ferrets were trained on an approach-to-target localisation task using similar methods to Parsons et al. [30]. Briefly, the ferret was first trained to lick the start spout at the centre of the chamber which initiated the presentation of a repeating stimulus (sound or light with duration of 1000 ms looped with a 500 ms gap). The ferret was then free to leave the start spout and respond at one of the response spouts at the periphery of the chamber. The trial continued with the stimulus repeating until the ferret made a correct response, whereupon it received a water reward. If the ferret left the central spout during the first stimulus presentation the trial was immediately terminated and the ferret would have to wait for 1 second before it could initiate the trial again. Once the ferret was accustomed to the nature of the task (identified by regular returning to the start spout after receiving water from target locations), a contingency was added whereby incorrect responses terminated the trial and the ferret was then required to initiate a new trial where it was presented with the same stimulus (correction trial) until a correct response was made. Over the course of a few weeks, timeouts were introduced for incorrect responses where the ferret was made to wait for a period of time before a new trial could be initiated. Timeout duration was increased until they lasted 5–7 seconds. Variable waiting times at the start spout before the presentation of the sound were also introduced during this training period such that the ferret would have to maintain contact with the start spout for a period of time before the stimulus was presented. These waiting periods gradually increased so that they were at least 500 ms and during testing, hold times of 500–1000 ms were used, pseudo-randomly selected on each trial. Once ferrets reached 60% correct or more at this stage, the stimulus was reduced to a single presentation (Fig 1B). Once the ferrets reached a performance level of ≥60% accuracy the stimulus duration was reduced to the next longest (500, 250 and 100 ms sound durations were tested). Ferrets were ready for testing at these durations once their performance stabilised (~3–4 weeks). Behavioural performance in our smaller testing chamber, which led to response spouts being located at half the distance of the speaker from the centre of the chamber, was lower than reported in previous studies in larger arenas [25]. For this reason we did not test with stimuli shorter than 100 ms in duration.

Two ferrets (F1202 & F1204) were initially trained and tested on the auditory localisation task before being trained to localise visual stimuli. For these ferrets, during visual training, stimuli were presented simultaneously with co-located auditory stimuli (1000 ms duration). Over several training sessions, the intensity of auditory stimulus was reduced and ultimately removed so that the ferret was localising the light source. Once the peripheral noise stimulus had been removed completely the ferrets appeared reluctant to move away from the start spout during presentation of the visual stimulus thus a noise stimulus of the same duration as the LED was re-introduced but was presented from above the ferret, where it offered no localisation cues. One ferret, F1204, could not reach a consistent level of performance on visual trials and therefore was not tested in the visual localisation task.

One ferret (F1311) was trained simultaneously on auditory and visual trials (randomly interleaved) using the methods described for sound localisation above. For this ferret, visual stimuli were placed at the same distance as the reward spouts as opposed to where the speakers were positioned (see Fig 1A). This animal required no additional acoustic stimulus from above. Table 1 summarises the testing each ferret completed.

**Table 1:** Ferret behaviour and cooling.

Behaviour		Auditory Localisation									Visual localisation		
Cooling		Left			Right			Bilateral			Left	Right	Bilateral
Duration (ms)		100	250	500	100	250	500	100	250	500	N/A		
Ferret	F1202	✓	✓	✓	✓	✓	✓					✓	
	F1204	✓	✓	✓	✓	✓	✓	✓					
	F1311	✓	✓		✓	✓		✓	✓		✓	✓	✓

### Surgical methods: Cooling loop Implantation

Anaesthesia was induced by a single dose of a mixture of medetomidine (Domitor; 0.022 mg/kg/h; Pfizer) and ketamine (Ketaset; 5 mg/kg/h; Fort Dodge Animal Health). The ferret was intubated, placed on a ventilator and ventilated with oxygen and isoflurane (1–3.5%) to maintain anaesthesia throughout the surgery. Supplementary doses of ketamine (where necessary) and saline were also given intravenously and local analgesic (Marcaine, 0.5%) injected under the skin prior to midline incision.

An incision was made along the midline of the ferret’s scalp and the connecting tissue divided from the skin to reveal the underlying temporal muscles. The posterior two thirds of each muscle were removed to expose the dorsal and lateral parts of the skull. Two holes for the bone screws located posteriorly and towards the midline of the animal were then drilled. Self-tapping bone screws were inserted into the skull to anchor the dental cement applied at the end of the surgery to secure the implant to the skull. A craniotomy over the ectosylvian gyrus was then made and the dura removed so that the cooling loop could be placed in direct contact with the brain.

A cooling loop was mounted on a micro-manipulator (Harvard Apparatus, USA) and carefully positioned over the dorsal tip of the ectosylvian gyrus on the MEG, aiming for A1. The boundaries of the cortical fields were not mapped prior to implantation to preserve the cortex and were instead confirmed with cytoarchitectonic markers post-mortem. The loop was lowered to directly contact the cortical surface and the craniotomy was sealed with Silastic (Kwik-Sil™, WPI Inc.) to protect the exposed brain. The loop and attached thermocouple (Fig 1C) were then secured in place with dental acrylic (Palacos R+G, Haraeus, Germany) and excess skin removed to create a tight wound margin around the implant. Further analgesia (Marcaine, 0.5%) was injected around the wound margin before the ferret was allowed to recover from the surgery. Ferrets were given post-operative analgesia (buprenorphine, 0.03–0.05 mg/kg), antibiotics (Amoxycare LA, 15 mg/kg) and anti-inflammatories (Loxicam, 0.05 mg/kg) for five days and allowed to recover for at least two weeks before recommencing behavioural testing.

### Cooling during behaviour

The cooling apparatus was adapted for use during behaviour by including an additional connector before the loop on the animal’s head. This allowed short pieces of tubing (0.5 x 50 mm and 0.5 x 200 mm, PTFE) to be attached to the loop while the ferret was distracted by animal treats (Nutri-plus gel, Virbac, UK). The tubing was then connected to the pump and ethanol reservoir.

For a cooling session, the apparatus was first ‘pre-cooled’ before connecting an animal by pumping ethanol through spare cooling loops until loop temperatures fell below 0°C. The animal was then connected to the system (Fig 1D) using the modified tubing and thermocouple connectors to monitor loop temperature at the cortical surface. The temperature was monitored online using a wireless transfer system (UWTC-1, Omega Engineering Ltd., Manchester, UK) and the flow rates adjusted to maintain a cortical temperature of ≤10°C (mean ± standard deviation (SD) across all cooled trials and ferrets, unilateral: 9.1 ± 0.8°C, bilateral: 8.7 ± 0.8°C). While waiting to reach the target temperature, the ferret was held outside the testing box and distracted with animal treats. When the target temperature range was reached, the animal was placed in the testing chamber to perform the localisation task.

Cooling sessions were performed in one of the twice-daily testing sessions of days 2–5 of a 5-day testing run. The other testing session from the same day was used as a warm control session. During these sessions the cooling apparatus was attached to the ferret but no cooling was performed. In order to maximise the number of trials obtained per cooling sessions, ferrets did not receive correction trials during the cooling sessions or the warm control sessions performed on the same day.

### Histology

When experiments were completed, ferrets were anaesthetised with a mix of ketamine and medetomidine followed by a terminal overdose of sodium pentobarbitone (2.5 ml/kg). Once deeply anesthetised the ferret was trans-cardially perfused with 0.9% saline followed by 4% paraformaldehyde. The brain was removed and placed in 4% paraformaldehyde overnight. The brain was then transferred to 30% sucrose (3–4 days) for cryo-protection prior to frozen sectioning at 50 μm in the coronal plane. Sections were collected in two series, one of which was subsequently stained with 0.2% Cresyl violet while the other was stained for neuro-filament H. For this we used the ABC procedure with DAB staining, briefly: All solutions were made with 10 mM PBS and Normal Horse Serum (NHS, Vector, S-2000) unless otherwise stated. Sections were initially incubated in blocking solution (5% NHS) for one hour and then overnight at 4°C with primary antibody (2% NHS, 1:4000 monoclonal mouse anti-Neurofilament H; BioLegend, San Diego, CA; mouse IgG1, cat# 801701, RRID: AB_2564642). After rinsing in PBS, sections were incubated with secondary antibody (2% NHS, 1:200 biotinylated horse anti-mouse IgG (H 1 L), Vector, cat# BA-2000, RRID: AB_2313581). After rinsing again in PBS, sections were incubation in ABC, rinsed and immunostaining visualized using DAB with nickel–cobalt intensification. Slides were imaged and digitized on an Axioscan Z1 slide scanner (Zeiss) and visualised using ZenLite software (Zeiss) in order to define the boundaries of the MEG and PEG/AEG [32] and identify the cooling loop location.

### Statistical analysis

To test the effect of cooling on activity of neurons in the suprasylvian gyrus of two anaesthetised ferrets, a two-way repeated measures analysis of variance (ANOVA) was performed in SPSS software (IBM SPSS, NY, USA) with firing rate as the dependent variable and cooling condition (pre-cooling, during cooling or post-cooling) and the cortical layer of the unit (layers I-IV or V-VI) as independent variables. Bonferroni post-hoc pairwise comparisons were made to compare the means (p < 0.05).

To assess the effect of sound duration on sound localisation performance in warm conditions, the response outcomes (correct / incorrect) at each duration for each ferret were fit by a generalised linear models (GLMs) assuming binomial distributions and using the logit link function (MATLAB R2014b; fitglm function). An effect of duration was found significant if inclusion of duration as a predictor term resulted in a significantly improved fit compared with the constant model (analysis of deviance, *χ*^*2*^ distribution, *p* < 0.05).

Effects of cooling on localisation accuracy were analysed separately for each ferret and for each stimulus modality (auditory/visual) by GLMs assuming binomial distributions and using the logit link function with response outcome (correct (1) / incorrect (0)) as the dependent variable. To determine whether cooling elicited any change in the unsigned error magnitude, generalised linear models were fitted to single subject data but under the assumption (given that errors could not be less than zero) that error magnitude followed a Poisson distribution and the log link function with error magnitude (on error trials) as the dependent variable.

To investigate effects on performance during unilateral cooling, models were fitted separately to data obtained when the left or right hemisphere was cooled. Each model tested the association between the animal’s response and four predictor terms: stimulus duration (500, 250, 100 ms), hemifield of the presented stimulus (left (0) / right (1)), cortical temperature (warm (0) / cooled (1)) and an interaction term between stimulus hemifield and temperature. The interaction between hemifield and temperature allowed us to test the hypothesis that unilateral cooling should only impair sound localisation in the contralateral hemifield and not the ipsilateral hemifield. The effect of unilateral cooling on midline performance was investigated by performing a fit with two predictor terms: stimulus duration (500, 250, 100 ms) and cortical temperature (warm / cooled).

To investigate the effects of bilateral cooling on performance across space, a single model was fitted to behaviour of each subject. For one subject tested with a single sound duration, (F1204, 100 ms) the model tested the effect of cortical temperature (cooled/warm). For the other subject (F1311), the model included both cortical temperature (cooled/warm) and stimulus duration (100, 250 ms). The significance of effects was assessed using a t-test against the null hypothesis that each coefficient was zero. For fitting visual data, the duration term was omitted since only one visual stimulus duration was tested.

For each model result we present the degrees of freedom, which indicates the number of trials in each test less the number of coefficients. We also present the exponential values of the estimated coefficients (*e*^*ß*^), which indicate the size of the effect of the variable on the dependent factor. For the logit link regression, *e*^*ß*^ indicates the change in odds of the dependent variable occurring (i.e. a correct response) with a change in predictor (i.e. going from warm to cooled). Values approaching one indicate no effect of the predictor on performance, whereas values above or below one indicate that changes in the predictor improved or impaired performance respectively. For the log-linked Poisson regression, *e*^*ß*^ gives the incidence rate ratio, the factor by which error magnitude would change given a single unit change in the predictor (e.g. increasing stimulus duration by 1 ms or for categorical predictors, going from warm (0) to cooled (1)) while holding all other variables constant. Values approaching one indicated no effect of the predictor, whereas values greater or less than one indicated increased / decreased error magnitude with a change in predictor. For each coefficient we also present the t-statistic and p value from a t-test against the null hypothesis that the coefficient was zero (and thus *e*^*ß*^ = 1). We took a p value of <0.05 to be significant.

## Results

### Determining efficacy of cooling: electrophysiology and temperature measurements in vivo

We first established that the cooling loop technique reduced cortical temperature by thermal mapping of auditory cortex in the anaesthetised ferret. A cooling loop was positioned over A1 and cooled to ≤10°C. Temperatures were then measured in cortex in the centre of the loop and surrounding areas at 5 depths in each location (500, 1000, 1500, 2000 and 2500 μm from the cortical surface, Fig 2A). Pumping chilled ethanol through the loop reduced cortical temperature and this decrease was rapidly attenuated as lateral distance on the cortical surface and/or depth from the loop increased (Fig 2B). At lateral distances further than 500 μm from the outside of the loop the temperature in cortex did not fall below 20°C (the level which is required for cessation of neural firing [5]); thus cooling was contained to within A1 (Fig 2B). Auditory cortex in ferrets is typically ~1500 μm thick reaching a maximum of 2000 μm only at the thickest part of the gyrus [28]. At 1500 μm in depth cortical temperature measurements from the centre of the loop were <20°C and remained so until ~1750 μm indicating that it was likely that all layers of the cortex were cooled in the centre of the loop. At all of the locations tested at a depth of 2500 μm the cortical temperature was greater than 20°C indicating that it was unlikely that cessation of neural firing occurred in other brain areas beneath A1.

In two further anaesthetised subjects with the cooling loop positioned over a visual cortical area on the surface of the suprasylvian gyrus (including areas 21 and PS) the LFP and the activity of units located in the middle of the cooling loop was recorded in response to 100 ms flashes of white light. Neural activity was recorded in visual cortex as stimulus-evoked responses under anaesthesia are more reliable than those in auditory cortex and elicit greater changes in firing rate [2]. Fig 3A shows a typical averaged light-evoked LFP from each electrode on the probe for one recording site before, during and after cooling. It is clear that the LFP is highly attenuated once the temperature of the cooling loop falls to <10°C. To estimate the depth/layer of each unit and to further understand the changes in cortical activity during cooling, CSD analysis was performed (Fig 3B). This revealed that there was attenuation of current sources and sinks during cooling with minimal activity when the loop was <10°C.

As the large attenuation of the LFP amplitude suggests, we found there was decrease in the firing rate of units across all depths of the cortex (Fig 3C–3F). Fig 3C shows the spiking responses from a typical multi-unit in which visually evoked responses before cooling are significantly attenuated during cooling and return to pre-cooling levels upon re-warming. The effects of cooling and its reversal occurred over several minutes and neural activity took longer to recover than fall (Fig 3D), consistent with previous cooling studies [10]. Across the population of units recorded (n = 72) cooling suppressed visually evoked spike rates (Fig 3E and 3F). Statistically this was significant as a main effect of cooling status (before cooling / cooled / after cooling) in a two-way repeated measures ANOVA (F_[2,64]_ = 51.51, p < 0.001). Pair-wise post-hoc analysis showed that firing rate during cooling for superficial (mean firing rate ± SD = 3.48 ± 10.2 spikes s^-1^) and deep layers (3.77 ± 7.4 spikes s^-1^) was significantly lower (p < 0.001) than before cooling (superficial: 66.5 ± 65.0 spikes s^-1^, deep: 57.24 ± 57.0 spikes s^-1^) and after cooling (p < 0.001, superficial: 52.5 ± 55.8 spikes s^-1^, deep: 45.4 ± 46.9 spikes s^-1^). However, recovery was not complete and the firing rate after recovery was still significantly less than before cooling (p<0.001). No effect of depth was observed on the firing rates of the units (F_[1,__32__]_ = 0.36, p = 0.553) or on the effect of cooling (depth * cooling condition, F_[2, 64]_ = 0.38, p = 0.689). Post-cooling firing rates were measured once the cortical temperature had returned to within 3°C of the pre-cooling temperature (25 – 71 minutes, mean ± SD = 54.5 ± 16 minutes). When a criterion for recovery of a return to within 20% of the pre-cooling maximum spike count was applied [13] 61/72 units met this criteria.

### Confirmation of cooling loop location in the chronic preparation

**Fig 4:**
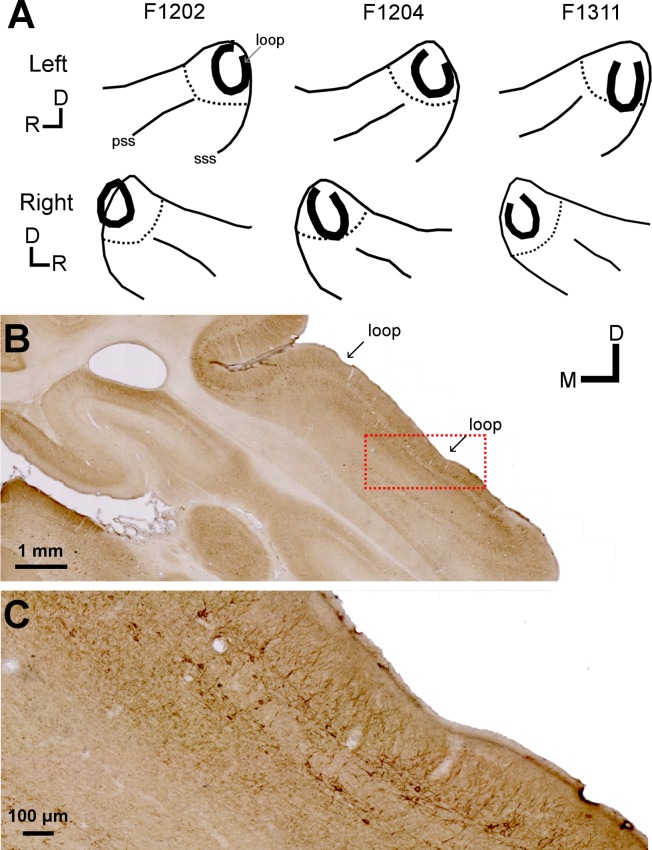
Histological verification of cooling loop locations. [A] Positions of cooling loops in each ferret (thick black lines). The auditory cortex is outlined in solid lines (sss: supra-sylvian sulcus, pss: pseudo-sylvian sulcus) with location of the Medial Ectosylvian Gyrus (MEG, where A1 is located) indicated by the dashed black lines, as determined by cytoarchitectonic boundaries derived from post-mortem histology). [B] Typical example of neurofilament stained brain section from underneath one of the loops (from F1204 right hemisphere). Cooling loop location indicated by arrows. Red dash box shows region of magnification in [C]. [C] Magnified image from [B] showing the cortex positioned beneath the loop is indistinguishable from elsewhere. Orientation axes: D = dorsal, R = rostral, M = medial.

Chronically implanted animals underwent histological processing after the completion of behavioural testing to confirm the location of the loops relative to anatomical and cytoarchitectonic boundaries [32] and to assess whether any gross changes to auditory cortex resulted from repeated cooling over a period of 7–15 months. After brain perfusion-fixation and removal, photographs of the loop location were taken and used to outline cooling loop position (Fig 4A). Each brain was then sectioned in order to determine the limits of MEG, where A1 and AAF are located, from Nissl and neurofilament stained sections (Fig 4B and 4C). In both large-scale images and brain sections, small indentations were visible immediately under the loops and these indentations, when combined with Nissl and neurofilament staining were used to confirm the position of the loops over A1. It is not possible to dissociate A1 and AAF cytoarchitectonically [32] and it is possible that the cooling loops silenced some of AAF in addition to A1, in particular in animal F1204 where the loops were positioned at a more rostral location relative to other animals. Other than the slight depressions on the cortical surface, the areas of brain underneath the loops were indistinguishable from other areas of cortex suggesting that there was no damage caused to the tissue underlying the cooling loops.

### Effect of cooling A1 on sound localisation performance

Having established through electrophysiological testing that cooling loop temperatures ≤10°C were sufficient to silence cortical activity in the ferret, and that the loops were accurately placed over the A1, we now consider the effect of reversible silencing on behaviour in a sound localisation task.

**Fig 5:**
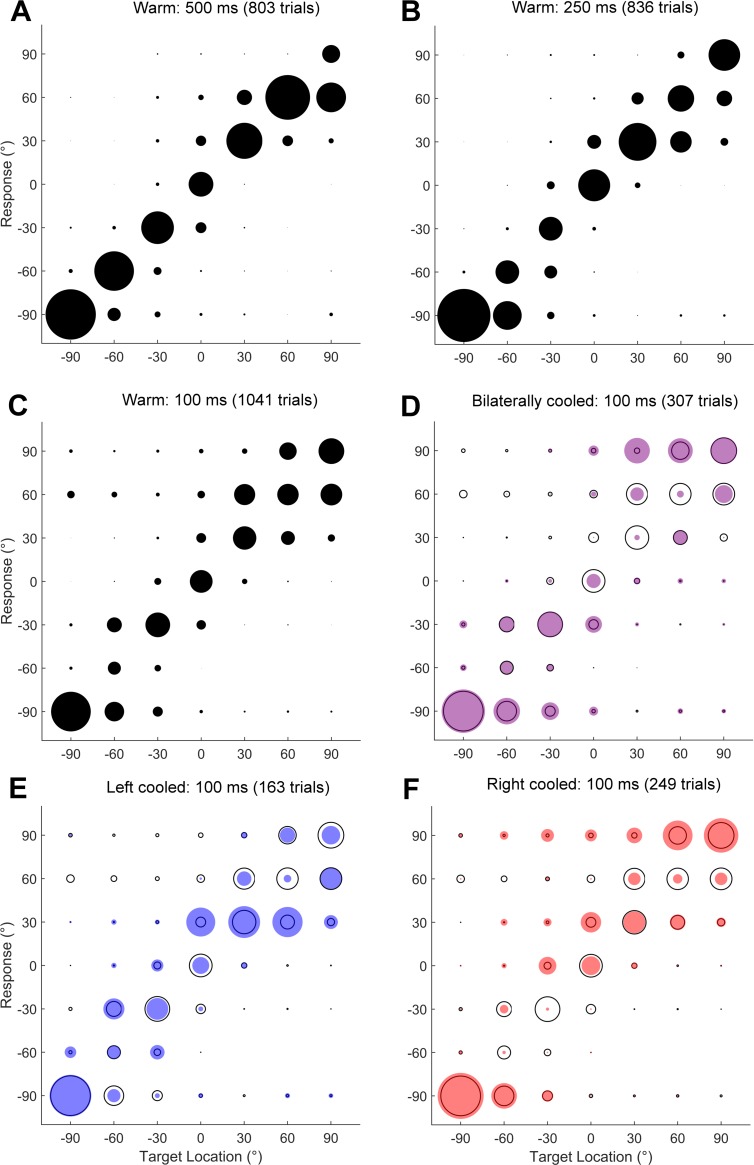
Sound localisation behaviour: an example ferret. [A–C] Responses of one ferret (F1204) to auditory stimuli of 500, 250 and 100 ms duration with the distribution of responses made to sounds at each location indicated by the filled circles. [D–F] Responses of the same subject to 100 ms sounds presented during bilateral (purple) and unilateral (left or right, blue or red respectively) cooling. Data from control sessions [C] shown in black outline for comparison. Negative locations indicate angles to the left of the midline.

**Fig 6:**
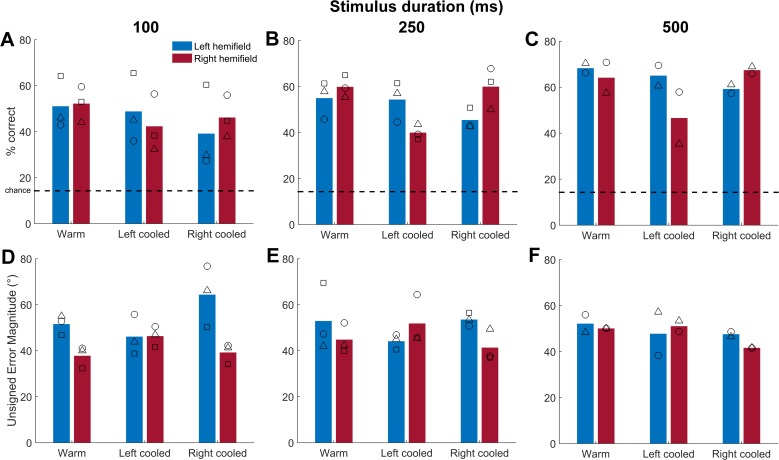
The effect of unilateral cooling on sound localisation performance in the left and right hemifields. [A—C] Mean performance across all ferrets (bars) and individual performance (symbols) for sound presented in left (-90°, -60° and -30°, blue) and right (90°, 60° and 30°, red) hemifields for warm control, left A1 cooled and right A1 cooled at each stimulus duration (indicated at the top of each column). Dashed line indicates chance performance. [D – F] Mean error magnitude for each condition stated above. Symbols: F1202 = circles, F1204 = triangles, F1311 = squares.

Prior to cooling loop implantation, ferrets were trained to perform an approach-to-target sound localisation task. In order to run animals tethered with cooling loop tubing we designed a smaller testing chamber that allowed approach-to-target localisation but minimised the distance animals had to move to reach the response spouts. In this smaller chamber, speakers were set back 8.5 cm from the response spouts (Fig 1A). Fig 5A–5C shows the data from one ferret for each target location at each of the stimulus durations tested, data from all animals is shown in Fig 6. Shortening the sound duration significantly reduced task performance in each ferret as revealed by a significantly improved GLM fit of response outcomes during warm conditions with inclusion of duration as a predictor term compared with the constant model (Analysis of deviance; F1202: *χ*^*2*^ (1659, N = 1661) = 27.24, p<0.001. F1204: *χ*^*2*^ (2488, N = 2490) = 47.17, p<0.001. F1311: *χ*^*2*^ (1707, N = 1709) = 8.37, p = 0.003) but even at the shortest duration performance remained above chance (chance: 14%; mean % correct ± SD at 100 ms: 47.9 ± 5.8%).

Animals were subsequently tested in the same localisation task with unilateral or bilateral cooling of the loops positioned over A1 to ≤10°C (mean ± SD across all cooled trials and ferrets, unilateral: 9.1 ± 0.8°C, bilateral: 8.7 ± 0.8°C) to investigate the effects of acute inactivation on sound localisation performance. Fig 5D–5F shows the consequences of inactivation on the performance of one ferret localizing 100 ms target sounds. Compared with the control sessions (Fig 5C, black outlines), bilateral cooling disrupts performance throughout space and unilateral cooling causes an increased error rate on the contralateral side of space.

To test the hypothesis that inactivating A1 elicits a contralateral deficit in sound localisation, behavioural performance was divided according to whether the target sound was presented at the midline or in the left or right hemifield. Performance within each region was then compared between warm control conditions and during unilateral cooling. Fig 6A–6C shows the performance of each ferret in the left and right hemispheres for each duration stimulus in warm, left cooled and right cooled conditions.

To determine whether cooling significantly influenced localisation behaviour, we fitted single trial responses of each animal with a generalised linear model that tested whether there was a significant association between the animal’s performance (correct / incorrect) and (1) cortical temperature (cooled vs. warm), which would indicate a general effect of cooling on performance, (2) the hemifield in which the stimulus was presented (left vs. right), which would indicate a difference in performance in each hemifield, (3) sound duration and (4) an interaction term between stimulus hemifield and cortical temperature, which would indicate a stronger effect of cooling in one hemifield over another. In this analysis, a contralateral deficit in sound localisation induced by cooling would be revealed as a significant interaction between hemifield and cortical temperature. Our GLM analysis enabled us to assess whether each of these factors were significant predictors of behavioural performance and to use the coefficients (see methods) to compare the direction and magnitude of each predictor term.

Across ferrets, hemispheres and durations, localisation performance decreased on average by 13.1% (F1202: -10.6%, F1204: -14.3%, F1311: -14.2%) in the contralateral hemifield and by 1.7% (F1202: -0.8%, F1204: -1.9%, F1311: -2.4%) in the ipsilateral hemifield during unilateral cooling compared with warm conditions. We confirmed the presence of a cooling-induced contralateral sound localisation deficit by demonstrating a significant interaction between cortical temperature and hemifield of sound for each ferret when cooling left A1 and for two of three ferrets when cooling right A1 (p < 0.05, Table 2). The coefficients were less than one when the left hemisphere was cooled and greater than one when the right hemisphere was cooled, signifying a contralateral effect of cooling on performance. As expected, the duration of the stimulus affected performance (Table 2, p<0.01). Temperature alone was a significant predictor only when the right hemisphere was cooled, not the left.

**Table 2:** Factors affecting sound localisation performance in GLM analysis for the left and right hemifields during unilateral cooling.

		Hemisphere cooled					
		Left			Right		
Subject		F1202	F1204	F1311	F1202	F1204	F1311
*df*		2268	2927	2064	2336	3031	1988
Sound Duration	*e*^*ß*^	1.0020	1.0018	1.0020	1.0020	1.0024	1.0024
	*t*	7.3565	7.8449	3.1500	7.4595	10.4270	3.8769
	*p*	1.9 x10^-13^	4.3 x10^-15^	0.0016	8.7 x10^-14^	1.9 x10^-25^	0.0001
hemifield of stimulus	*e*^*ß*^	1.5652	0.7706	0.7121	1.5647	0.7675	0.7117
	*t*	4.2500	-3.0663	-3.2502	4.2491	-3.0903	-3.2517
	*p*	2.1 x10^-5^	0.0022	0.0012	2.1 x10^-5^	0.0020	0.0011
Temperature	*e*^*ß*^	0.9474	0.8836	1.0301	0.7003	0.5894	0.6662
	*t*	-0.4201	-0.9659	0.1992	-2.8460	-4.2851	-2.6129
	*p*	0.6744	0.3341	0.8421	0.0044	1.8 x10^-5^	0.0090
Temperature * Hemifield	*e*^*ß*^	0.6122	0.6094	0.4783	1.5212	1.7940	1.3514
	*t*	-2.6914	-2.7074	-3.5314	2.3306	3.3970	1.3898
	*p*	0.0071	0.0068	0.0004	0.0198	0.0007	0.1646

We next asked for each ferret whether cooling increased the magnitude of errors on incorrect trials (Fig 6D–6F). Across duration and ferrets, average unsigned error magnitude increased in the hemifield contralateral to cooling by 4.6° (F1202: +6.7°, F1204: +5.7°, F1311: +1.3°) and decreased in the ipsilateral hemifield by 5.2° (F1202: -6.1°, F1204: +0.1°, F1311: -9.6°). To test this statistically we fitted unsigned error magnitudes on each error trial with the same model design as described above for single trial responses, though here we accounted for the minimum error boundary (30°) by assuming a Poisson distribution and using the log link function. In general, all factors in the model, including cooling, showed effects on the error magnitude of the ferrets (Table 3). The temperature-hemifield interaction term coefficients were greater than one when the left was cooled and less than one when the right was cooled, consistent with an increase in error magnitude in the hemifield contralateral to cooling.

**Table 3:** Factors affecting unsigned error magnitude during sound localisation in GLM analysis for the left and right hemifields during unilateral cooling.

		Hemisphere cooled					
		Left			Right		
Subject		F1202	F1204	F1311	F1202	F1204	F1311
*df*		1016	1405	878	1024	1443	824
Sound Duration	*e*^*ß*^	1.0000	1.0001	1.0020	0.9998	0.9996	1.0021
	*t*	-1.3376	-3.5645	28.8600	-6.5670	-14.7050	29.7160
	*p*	0.1810	0.0004	3.8 x10^-183^	5.1 x10^-11^	6.0 x10^-49^	4.8 x10^-194^
hemifield of stimulus	*e*^*ß*^	0.9400	0.8139	0.8382	0.9423	0.8181	0.8400
	*t*	-5.4811	-23.5500	-14.8530	-5.2617	-22.9690	-14.6600
	*p*	4.2 x10^-8^	1.3 x10^-122^	6.7 x10^-50^	1.4 x10^-7^	9.5 x10^-117^	1.2 x10^-48^
Temperature	*e*^*ß*^	0.9405	0.9061	0.7724	1.1856	1.0424	0.9898
	*t*	-4.7161	-7.3467	-14.0800	15.2460	3.6965	-0.6347
	*p*	2.4 x10^-6^	2.0 x10^-13^	5.0 x10^-45^	1.7 x10^-52^	0.0002	2.6 x10^-21^
Temperature * Hemifield	*e*^*ß*^	1.2755	1.2309	1.3118	0.7126	0.9640	0.8186
	*t*	13.3140	11.6330	11.5850	-17.7150	-2.1584	-8.2127
	*p*	1.9 x10^-40^	2.8 x10^-31^	4.9 x10^-31^	3.2 x10^-70^	0.0309	2.2 x10^-16^

**Fig 7:**
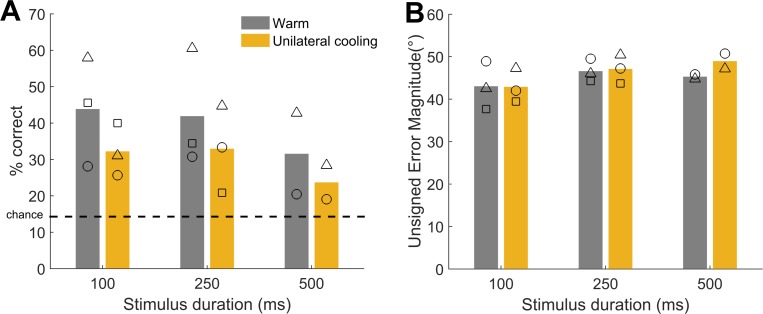
The effect of unilateral cooling sound localisation performance at the midline. [A] Mean performance at the midline for each stimulus duration for warm (grey) and unilaterally cooled (yellow) conditions. [B] Mean unsigned error magnitudes for each stimulus duration in warm and unilaterally cooled conditions. Symbols: F1202 = circles, F1204 = triangles, F1311 = squares.

We next addressed the effects of unilateral cooling on sound localisation at the midline both in terms of percentage correct (Fig 7A) and unsigned error magnitude (Fig 7B). Across ferrets and durations, on average there was a 9.7% decrease (F1202: +0.4%, F1204: -19.0%, F1311: -9.6%) in performance at the midline during cooling compared with warm conditions. We tested the prediction that performance at the midline would not be affected by unilateral cooling by fitting a GLM to single trial response outcomes with stimulus duration and cortical temperature (warm/cool) as predictors. Since there was no prior reason to expect differences between cooling of left and right hemispheres, we included trials from both data sets. We found a significant effect of cooling on midline localisation performance in only one of three ferrets (p < 0.05, Table 4). We found significant effects of cooling for two of three ferrets on the error magnitude, and a significant effect of duration in all ferrets (p<0.05). However, the sign of change for error magnitude varied among ferrets indicating that changes were inconsistent between the ferrets.

**Table 4:** Factors affecting midline sound localisation performance in the GLM analysis for the midline location during unilateral cooling.

Metric		Accuracy (correct/ incorrect)			Unsigned Error Magnitude		
Subject		F1202	F1204	F1311	F1202	F1204	F1311
*df*		512	623	403	373	363	261
Sound Duration	*e*^*ß*^	0.9987	0.9999	0.9952	1.0001	0.9998	1.0008
	*t*	-1.9725	-0.2338	-3.2271	2.8489	-4.2665	6.2766
	*p*	0.0486	0.8151	0.0013	0.0044	2.0 x10^-5^	3.5 x10^-10^
Temperature	*e*^*ß*^	0.9362	0.6548	0.8097	0.9872	1.0310	0.9166
	*t*	-0.3303	-2.4593	-0.9683	-0.8555	1.9617	-4.5575
	*p*	0.7412	0.0139	0.3329	0.3923	0.0498	5.2 x10^-6^

**Fig 8:**
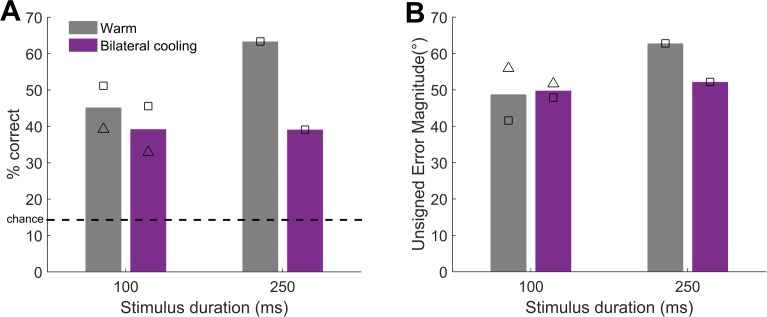
The effect of bilateral cooling on sound localisation performance. [A] Bars show the mean performance across all locations tested for each stimulus duration for warm and bilaterally cooled conditions. [B] Bars show the mean unsigned error magnitude for each stimulus duration for warm and bilaterally cooled conditions. Symbols: F1204 = triangles, F1311 = squares.

In two ferrets we also performed bilateral cooling (Fig 8) in which we predicted a deficit in sound localisation throughout space. Across ferret and durations, there was a decrease of 10.6% (F1204: -6.3%, F1311: -14.9%) in performance during bilateral cooling compared with warm conditions. We fitted GLMs to each subject’s behaviour and assessed the association between performance and cortical temperature (cooled/warm). In one subject we tested the effects of bilateral cooling on the localisation of sounds of varying durations (100 and 250 ms only) and thus also included sound duration in the model for that ferret. In both animals there was a significant effect of bilateral cooling (Table 5). Bilateral cooling also affected the magnitude of subjects’ errors although the effects were different between the two ferrets (Table 5, Fig 8B).

**Table 5:** Factors affecting sound localisation performance in GLM analysis for bilateral cooling.

Metric		Accuracy (correct/ incorrect)		Unsigned Error Magnitude	
Subject		F1204	F1311	F1204	F1311
*df*		1346	2356	808	1102
Sound Duration	*e*^*ß*^	N/A	1.0011	N/A	1.0017
	*t*	N/A	1.9020	N/A	29.1710
	*p*	N/A	0.0572	N/A	4.6x10^-187^
Temperature	*e*^*ß*^	0.6777	0.5744	0.9719	1.0556
	*t*	-2.8455	-5.7119	-2.5518	5.6852
	*p*	0.0044	1.1x10^-8^	0.0107	1.3x10^-8^

### Effect of cooling A1 on approach-to-target visual localisation performance

**Fig 9:**
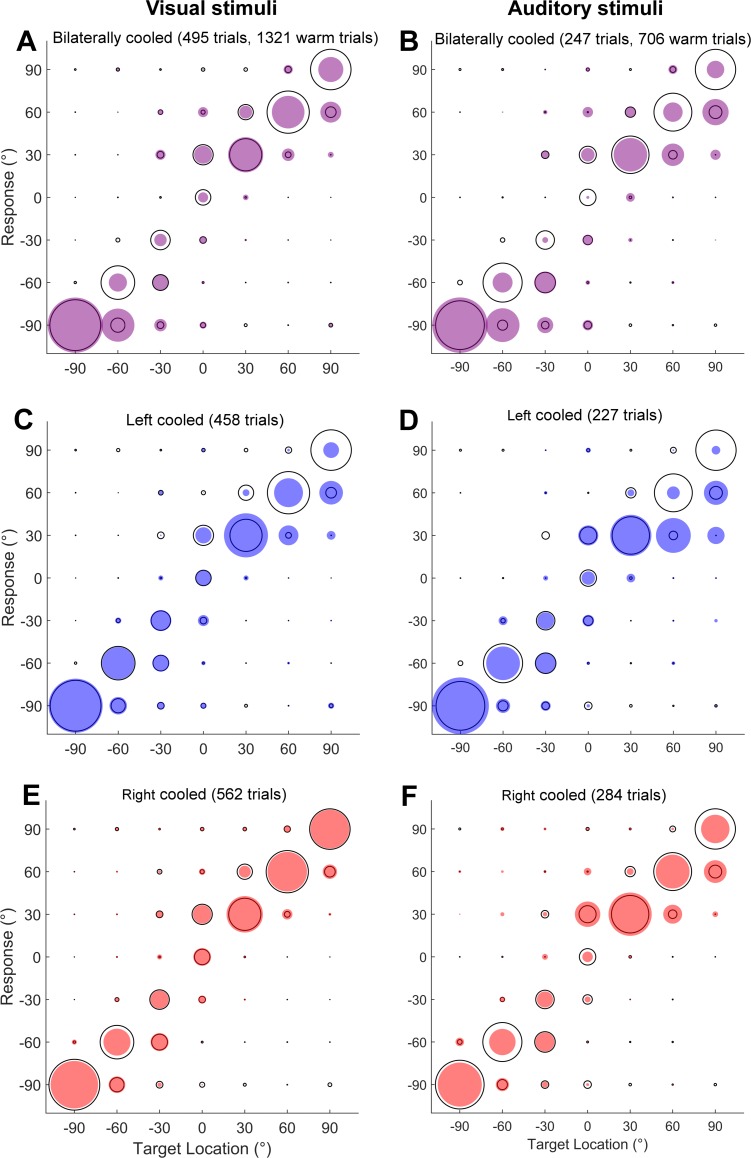
Comparison of the effects of cooling on visual and auditory localisation performance. The responses of ferret F1311 to visual stimuli (left column) and matched difficulty auditory stimuli (right column) during bilateral (A & B), left (C & D) and right (E & F) unilateral cooling with the warm performance shown in grey for comparison. The distribution of responses made to sounds at each location is indicated by the filled circles.

To show that the cooling induced deficits in sound localisation were specific to the auditory domain and did not generalize to other sensory modalities or result from general changes in motivation or locomotion, ferrets were also trained to approach the location of visual stimuli. We matched the difficulty of auditory and visual localisation by adjusting the duration of the light so that performance was equivalent to that observed when animals localized sounds of 250 ms in duration. Matching of stimulus difficulty produced similar performance in auditory and visual tasks under warm control conditions (Fig 9). However during cooling of A1, deficits in task performance were limited to auditory and not visual localisation (Tables 6–8).

**Table 6:** Factors affecting visual localisation performance and unsigned error magnitude in GLM analysis for the left and right hemifields during unilateral cooling.

Metric		Accuracy (correct/ incorrect)			Unsigned Error Magnitude		
Hemisphere cooled		Left	Right		Left	Right	
Subject		F1311	F1202	F1311	F1311	F1202	F1311
*df*		719	547	757	259	258	272
hemifield of stimulus	*e*^*ß*^	1.5664	0.9258	1.5664	0.9258	1.5707	0.9258
	*t*	2.4649	-0.2775	2.4649	-4.0818	19.4700	-4.0818
	*p*	0.0137	0.7814	0.0137	4.5x10^-5^	2.0x10^-84^	4.5x10^-5^
Temperature	*e*^*ß*^	1.2456	1.2060	0.9949	0.8058	1.1254	0.8505
	*t*	0.9058	0.7556	-0.0227	-8.1829	5.1468	-7.0169
	*p*	0.3650	0.4499	0.9819	2.8x10^-16^	2.6x10^-7^	2.3x10^-12^
Temperature * Hemifield	*e*^*ß*^	0.7733	1.2112	1.2616	1.4741	0.8296	0.8729
	*t*	-0.7346	0.5436	0.6983	10.4990	-6.2375	-3.5318
	*p*	0.4626	0.5868	0.4850	8.7x10^-26^	4.4x10^-10^	0.0004

**Table 7:** Factors affecting visual localisation performance and unsigned error magnitude in GLM analysis for the midline location during unilateral cooling.

Metric		Accuracy (correct/ incorrect)		Unsigned Error Magnitude	
Subject		F1202	F1311	F1202	F1311
*df*		92	163	49	112
Temperature	*e*^*ß*^	0.5773	1.7354	0.8608	1.0444
	*t*	-1.2200	1.6228	-3.8674	1.5567
	*p*	0.2225	0.1046	0.0001	0.1195

**Table 8:** Factors affecting visual localisation performance and unsigned error magnitude in GLM analysis for bilateral cooling.

Metric		Accuracy (correct/ incorrect)	Unsigned Error Magnitude
Subject		F1311	
*df*		861	371
Temperature	*e*^*ß*^	0.5773	1.5773
	*t*	-0.7304	-8.1217
	*p*	0.4652	4.6x10^-16^

**Fig 10:**
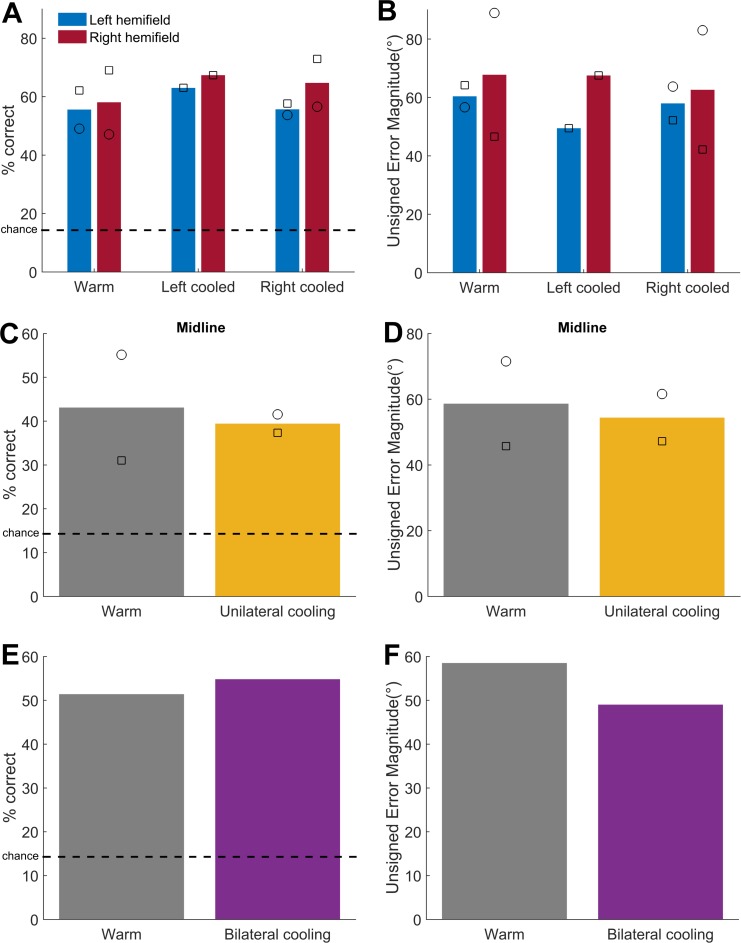
Effects of cooling on visual localisation performance. [A] Mean visual localisation performance and [B] change in error magnitude of the ferrets (bars: mean, symbols: individual animal mean) in each cooling condition for the left (blue) and right (red) hemifields [C] Mean performance and [D] error magnitude at the midline during warm (grey) and unilateral cooling (yellow) conditions. [E] Mean performance and [F] error magnitude of ferret F1311 across space during warm and bilateral cooling (purple) conditions. Symbols: F1202 = circles, F1311 = squares.

When addressing visual localisation specifically for each subject tested (Fig 10), we observed no systematic impairment in performance or error magnitude. To test this statistically we repeated our GLM analysis of single trial performance during cooling of right (2 ferrets) or left A1 (1 ferret), this time using a model with three predictors: hemifield of the visual stimulus, cool/warm condition and an interaction term between hemifield and warm/cool condition. Only one stimulus duration was used and thus duration was excluded from the model previously used for analysis of sound localisation. There were no significant effects of any term in either animal when either left or right hemisphere was cooled (p > 0.1, Table 6). However, for error magnitude, there was an effect of cooling in both animals and also temperature-hemifield of stimulus interaction in both animals. The sign of the interaction coefficient effect was opposite for left and right cooled hemispheres and was also opposite to those observed in sound localisation indicating that error magnitude actually decreased in the contralateral hemifield during unilateral cooling, also evident in Fig 10B.

We also tested the effect of unilateral cooling on midline performance for visual localization but found no significant effect of cooling for either subject tested (p > 0.1, Table 7). One of the two ferrets showed a decrease in the error magnitude (Table 7). For bilateral cooling in one subject, there was also no effect of bilateral cooling on visual localisation performance (Table 8). There was however a significant effect of cooling on error magnitude, indicating a decrease in error magnitude during cooling. Thus visual localisation performance during cooling of A1 was unimpaired by cooling, in contrast to the detrimental effect on performance and increased error magnitude during sound localisation.

## Discussion

The data presented here demonstrate cooling is a viable technique for selectively and reversibly inactivating auditory cortex in the behaving ferret as cooling related performance deficits were observed only in auditory and not visual localisation. Our experiments determining the spread of cooling and the consequences of cooling on neuronal activity suggest that behavioural impairment resulted from localized silencing of neuronal activity in A1.

The deficit caused by inactivation of A1 is consistent with results observed after other forms of inactivation in the ferret; unilateral pharmacological inactivation of A1 caused a contralateral localisation deficit [2], and bilateral inactivation by pharmacological means or lesion caused a deficit in sound localisation ability across space [2,24]. In contrast to bilateral pharmacological deactivation [2], where localisation deficits were only observed with 40 ms stimuli, both bilateral lesions [24] and inactivation by cooling resulted in deficits in localisation of 100 ms stimuli. For such 100 ms duration stimuli, deficits in performance observed during bilateral cooling (a drop in 6% across both subjects) were similar in magnitude to those resulting from bilateral ablation of A1 (approx. 10%) [24]. Such results are consistent with mechanisms of inactivation in which cooling and lesioning suppress all neural activity in the target region whereas pharmacological deactivation affects specific neuronal subtypes.

As in the cat [7,23], inactivation of A1 did not result in any increase in the number or size of the errors that animals made in localising visual stimuli. In fact in some instances, cooling A1 caused a decrease in the average size of the errors on those trials in which the target was mis-localised. It is possible that auditory cortex could modulate visual localisation directly or indirectly [33,34]. In comparison with the cat [7,23], cooling-related deficits in sound localisation in the ferret were modest (cooling induced change in performance contralateral to unilateral cooling: cat = 45.1%, ferret = 13.1%; bilateral cooling: cat = 47.8%, ferret = 10.6% [23]), however the changes observed were similar to those observed in previous lesion studies of the role of A1 in sound localisation in ferrets [24]. Smaller effects of cooling in the ferret may also, in part, be due to differences in the experimental designs used with different species: Cats were given the option to select a guaranteed ‘lower value’ reward by approaching the 0° position rather than the target stimulus location whereas ferrets were required to respond regardless of their certainty about the sound’s location. This may have encouraged a cleaner pattern of errors since if the cats were uncertain about the target location they had the option to select a guaranteed reward. Furthermore, Malhotra and colleagues [7,23] cooled the cat brain to 3 ± 1°C, approximately 6°C lower than in the present study. We selected our temperature based on (1) our observation that cooling the cortical surface below 10°C significantly reduced cortical activity in the anaesthetised ferret (Fig 3) and (2) our observations that cooling the cortical surface to 10°C did not induce decreases in cortical temperature below 20°C at depths beyond the thickness of ferret cortex (1.5–2 mm, Fig 2B) nor at lateral locations outside of A1 (see locations 8, 10 and 11 in Fig 2).

Spread of cooling was a particular concern as use of low temperatures in small mammals may lead a drop in temperature in other brain areas and even the cochlea [10], Coomber and colleagues [10] cooled the surface of the guinea pig brain to 2°C and demonstrated a small temperature drop (~4°C) in the thalamus, midbrain and middle ear, but this small change was not sufficient to directly reduce neural activity. However it has been shown in birds that lowering the temperature of cortex to levels above those required for cessation of neural firing can slow down neural firing rates [8]. Although we did not test other brain areas, the cortical temperature at the deepest locations measured (2500 μm) did not drop below 20°C with the cooling loop temperature at 10°C, thus is it unlikely that any other brain areas were inactivated but it is possible that some areas were affected by a temperature drop that was not sufficient to alter neural firing rates.

When considering the extent of cortex inactivated during behaviour in the present study, neurons in most cortical layers were likely attenuated by cooling but only within the area under the loop. Fig 3 indicates that there was a significant decrease in firing rate of cortical units from deep layers V-VI during cooling and that this was not different from the reduction achieved in the more superficial layers (Fig 3F), thus it is likely that we achieved inactivation of all layers of cortex during behaviour. This is consistent with work in anaesthetised guinea pigs showing cooling the cortical surface to 2°C reduced the temperature at 2 mm cortical depth to less than 20°C [10]. While warmer than at the cortical surface, cooling deep layers to this temperature is still likely to suppress neural activity [5,10,35]. Selecting a temperature at which to cool is necessarily a balance between successfully inactivating the whole cortical depth (1.5–2 mm in ferrets) and restricting the spread of cooling to under and within the area covered by the cooling loop. Fig 2 demonstrates that with a surface temperature of 10°C, the spread of cooling laterally outside of A1 was minimal and that deeper than the thickness of auditory cortex, no temperatures dropped below 20°C. Indeed given that in anesthetized animals the cortex is exposed rather than protected and therefore not insulated as in chronically implanted, freely moving animals, our measurements of cooling spread are likely to overestimate the spread occurring in behaving ferrets.

During our testing of the effect of cooling on neural data, we observed a significant decrease in firing rate during cooling in superficial (I-IV) and deep layers (V-VI) compared with before cooling. We observed only partial recovery of units to pre-cooling levels, and although post-cooling firing rates were significantly different from rates during cooling, the post-cooling levels were significantly different to pre-cooling. There are many reasons why neurons may not have fully recovered their pre-cooling firing rates including not enough time being left for recovery and/or a lack of firing/input leading to changes in adaptation in the neurons in question. Applying a criteria for recovery defined by Antunes and Malmierca [13] as a recovery of spiking to >80% of the pre-cooling maximum the majority (62/72) of neurons recovered. Furthermore, we did not observe any prolonged deficit in ferret performance of localisation after cooling, thus is unlikely that there was any prolonged impairment of cortical processing through cooling.

In summary, this work supports the successful application of inactivation of auditory cortex by cooling as assessed by performance impairment in a sound localisation task. Consistent with previous work in ferrets [2,20,24,25] and other species including primates and carnivores [17,18,21,22,36], unilateral inactivation of A1 resulted in a contralateral localisation deficit and bilateral inactivation resulted in deficits across space. Cooling did not affect visual localisation performance in the same task design, demonstrating that behavioural impairments were not related to non-specific effects on motivation and motor coordination. Recordings of cortical temperature and neural activity in anesthetized subjects suggest that cooling was specific to the vicinity of the loop over auditory cortex and behavioural deficits stemmed from a suppression of neural firing in this region. Our results establish cooling as a viable technique in the ferret, offering a method for cortical inactivation in a range of other psychophysical tasks (e.g. [37]) for which ferrets are an excellent model in behavioural neuroscience.

## Supporting Information

S1 File Data underlying each figure. Each tab shows data underlying figures presenting results.
(XLSX)
